# Study of treatment using percutaneous acetabuloplasty and interstitial implantation of ^125^I seeds for patients with metastatic periacetabular tumors

**DOI:** 10.1186/1477-7819-10-250

**Published:** 2012-11-20

**Authors:** Jinlei Zhang, Zuozhang Yang, Jiaping Wang, Jinde Wang, Pengjie Liu, Hongpu Sun, Kun Li, Yunshan Yang

**Affiliations:** 1 Department of Orthopedics, The Third Affiliated Hospital of Kunming Medical University, Tumor Hospital of Yunnan Province, Kunming, 650118, P.R. China; 2 Department of Radiology, The Second Affiliated Hospital of Kunming Medical University, Kunming, Yunnan, 650118, P.R. China; 3 Kunming Medical University, Kunming, Yunnan, 650118, P.R. China; 4 Department of Nuclear Medicine, The Third Affiliated Hospital of Kunming Medical University, Tumor Hospital of Yunnan Province, Kunming, 650118, P.R. China; 5 Department of Radiology, The Third Affiliated Hospital of Kunming Medical University, Tumor Hospital of Yunnan Province, Kunming, 650118, P.R. China; 6 Department of intervention therapy, The Third Affiliated Hospital of Kunming Medical University, Tumor Hospital of Yunnan Province, Kunming, 650118, P.R. China

**Keywords:** Periacetabular, Metastatic tumor, ^125^I seed implantation, Percutaneous acetabuloplasty, Bone cement

## Abstract

**Background:**

The periacetabular area is one of the primary sites of metastatic tumors, which often present as osteolytic bone destruction. Bone destruction in the acetabulum caused by metastatic tumors will cause hip pain and joint dysfunction. It results in decreased quality of life for patients. The aim of our study was to explore the clinical effect of metastatic periacetabular tumors treated with percutaneous cementoplasty and interstitial implantation of ^125^I seeds.

**Methods:**

A retrospective analysis was performed on 24 patients with metastatic periacetabular tumors who underwent combined therapy of percutaneous acetabuloplasty and interstitial implantation of ^125^I seeds between February 2003 and June 2011. There were 13 males and 11 females aged 19–80 years with a mean age of 57.3. The primary tumor site was the lung in eight cases, the breast in six, the prostate cancer in eight, and the liver in two. The amount of implanted ^125^I seeds was 12–20 seeds/person, with a mean of 16.5 seeds/person, and the matching peripheral dosage (MPD) was 80~100Gy. Routine postoperative chemotherapy and other combined treatments were applied to patients after the surgical operation. Changes in the Karnofsky Score(KPS), Harris Hip Score(Harris), and Visual Analog Scale(VAS) were observed during the follow-up period.

**Results:**

The 24 patients’ operations were all successful. No major complications occurred. Complete pain relief was achieved in 58% (14 of 24) of patients, and pain reduction was achieved in the 42% remaining (10) patients. The mean duration of pain relief was 8.3 months. Pain recurred in one patient 3 months after surgery. Six patients had died and 18 patients were alive at the time of the 1-year follow-up. Comparing the KPS, Harris and VAS scores pre- and postoperativelyat 1, 6, and 12 months, the combined therapy method was significantly effective in metastatic periacetabular tumor patients (*P*<0.05).

**Conclusions:**

Percutaneous cementoplasty with interstitial implantation of ^125^I seeds is an effective treatment method for metastatic periacetabular tumor patients, providing tumor resistance, pain relief, increased bone stability, and improved quality of life for patients.

## Background

The periacetabular area is one of the primary sites of metastatic tumors, which often present as osteolytic bone destruction. Bone destruction in the acetabulum caused by metastatic tumors causes hip pain and joint dysfunction, resulting in decreased quality of life for the patients [1].

Radiotherapy and surgery are the commonly used methods to treat acetabular metastases. Radiotherapy achieves pain relief rate in 75% or more of the patients with metastatic tumors, but marked clinical improvement is observed in all patients 1 or 2 weeks after radiotherapy. However, radiotherapy cannot cure structural weakening of the pelvis caused by tumors and also leads to regional osteoporosis caused by radiotherapy, which increases the incidence of pathological fractures [2,3]. Harrington and colleagues [4] showed that hip reconstruction for periacetabular metastasis could improve the functional status and reduce pain. For reconstructions of the hip joint and bone defect repairs, maintaining the normal lines of force needs to be considered when operating. Because of the poor general condition of patients, complexity of the operative process, and large wounds, reconstruction can involve the complications of local recurrence, deep infections, dislocations, and fractures of the internal implant in the perioperative period [5-11]. Extensive resection and reconstruction usually bring more risks than advantages and are not the preferred operative option [12]. Instead, tumor curettage and bone cement sometimes achieve better results.

Percutaneous acetabuloplasty is the injection of acrylic bone cement into malignant or benign bone cavities in order to relieve pain and/or stabilize the bone. Bone packing with cement aims to treat or prevent vertebral and extraspinal pathological fractures and relieve pain in patients with osteoporosis and bone metastases, which, if applied in the treatment of acetabular metastases after surgery, could effectively improve the patients' quality of life [13-15].

^125^I particle implantation, as an effective interstitial brachytherapy (IBT) technique, has been widely reported recently. Interstitial brachytherapy refers to the implantation of radioactive sources, usually in the form of needles, seeds, or wires, which are directly injected into the tumor lesion. Radioactive seed implantation has the advantages of real-time monitoring, precise orientation, being a simple operation, causing no radiation injury, etc., which can efficiently improve local control or increase the survival rate of patients with bone metastases [16-18]. The aim of this technique is to tailor the dose of irradiation to the anatomy of the patient for a better target volume coverage.

The aim of our study was to evaluate the efficacy of percutaneous puncture of the bone cement combined with ^125^I seed implantation in the treatment of 24 patients with acetabular metastases enrolled from February 2003 to June 2011.

## Methods

### Patients

Twenty-four patients with periacetabular metastatic tumors were treated using percutaneous puncture of the bone cement combined with^ 125^I particle implantation from February 2003 to June 2011. We obtained approval for the study from the Ethics Committee of Tumor Hospital of Yunnan Province. The subjects and their families were well informed of the details and signed relevant contracts prior to the study. Patient ages ranged from 18 to 80 years; follow-upduration ranged from 8 to 60 months. There were single periacetabular metastases in 8 patients and and multiple bone metastases accompanied by periacetabular metastases in 16 patients. In 6 cases the primary tumors metastasized after resection in 6 months to 7 years; in 13 patients the primary tumor metastasized after radiotherapy and biological treatment within about 6 months to 2 years. Pain from the first metastasis occurred in five patients, and the primary malignancy site was found after inspection. The demographic data and primary malignancy site are shown in Table 1.

**Table 1:** Baseline characteristics of the patients

**Parameters**	
No. of patients	
Male	24
Female	13
Median age (years)	11
Median follow-up duration (months)	57.3±3.52
Site of primary malignancy	39±4.32
Lung	8
Breast	6
Prostate	8
Liver	2

Participants were patients with the following conditions: (1) the pathology had been confirmed by cytology in malignant tumor patients; (2) expected survival time of over 3 months; (3) persistent periacetabular pain, and no significant improvement achieved after medication and/or physical therapy. They also had confirmed periacetabular osteolytic destruction after X-ray, CT, and MRI imaging assessment.

Indications and contraindications for percutaneous acetabuloplasty and ^125^I seed implantation according to Maccauro [19] are detailed in Table 2.

**Table 2:** **Indications and contraindications for percutaneous acetabuloplasty and**^**125**^**I seed implantation**

**Indications**	**Contraindications**
Weight-bearing periacetabularosteolysis	**1: Absolute contraindications**
Hip pain resistant to drugs	Acetabular fracture
Patients with multiple metastases	Pelvic discontinuity
Life expectancy ≥3 months	**2: Relative contraindications**
Inability to tolerate major surgery	Radiographic signs of medial wall interruption
Ineffective radiotherapy	Local infection
	Hemorrhagic disorders

### Instruments and radioactive source

A domestic bone cement valvuloplasty device was used in our evaluation; the device included a puncture needle and a precession type injector pressure device (Guan Long Company, Shandong, China). A closed radioactive isotope ^125^I source was selected as the radioactive source (China Institute of Atomic Energy Isotopes, Beijing;Chinese Drug Approval No. H20045969). The radiation source is cylindrical, and the diameter and height are 0.8 mm and 4.5 mm, respectively, with tissue-penetrating ability of 1.7cm and a half value layer of 0.025 mmPb. It is surfaced with titanium alloy inclusions, with a source activity of 0.3−1 mci. Liquid immersion disinfection was selected using benzalkonium bromide (soaked in Bromogeramine solution for 30 min).

### Treatment planning system

The treatment planning system was provided by Nuclear Industry Corp., Beijing Kelinzhong Medical Technology Institute. CT/MRI image scan results were obtained with three-dimensional digital image reconstruction in each patient before treatment using the system software according to lesion size, location, and the relationship with surrounding normal tissues. Three-dimensional icons and isodose curves were drawn to indicate the precise formulation and absorbed dose. At the same time, the radiation source initial dose, applicator needle coordinate, and depth indicator were given, and the treatment plan form was printed out. The initial dose of ^125^I particles was 2.92cGy/h/particles, and the 90% isodose curve included 90% tumor target volume. The tumor periphery matching dose (MPD) was 80 to100Gy.

### Surgical technique

**Figure 1:**
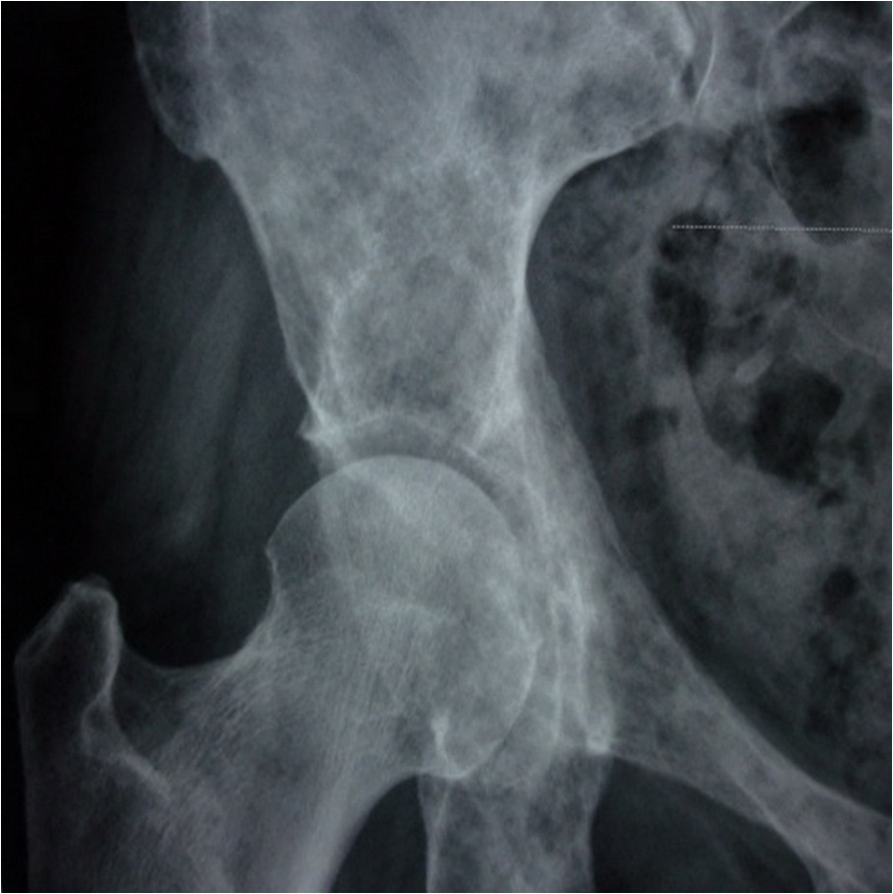
Right margin of the acetabulum in metastatic breast cancer before the operation.

Routine tests of preoperative cardiopulmonary function, blood glucose, blood coagulation, liver and kidney function, and iodine allergy were conducted before the operation. The extent of lesions was determine by X-ray, CT, or MRI examination (Figure 1) to choose the optimal operation line. The interventional operations were conductedusingDSA machine guidance under sterile conditions; all patients received analgesic treatment 15 min before the operation.

### Steps

Step 1: A lateral patient position was required to confirm the lesion locationusing DSA. The needle was disinfected with 1% lidocaine. The puncture needle was always perpendicular to the cortical bone destruction area in order to facilitate the puncture.

Step 2: The puncture needle was inserted into the bone fracture zone in an open perspective, then was pulled out. ^125^I particles were implanted according to the preoperative TPS treatment planning with an injection distance of 0.3 cm. A beveled needle and changed needle puncture direction were used for optimal implantation of the ^125^I particles.

**Figure 2:**
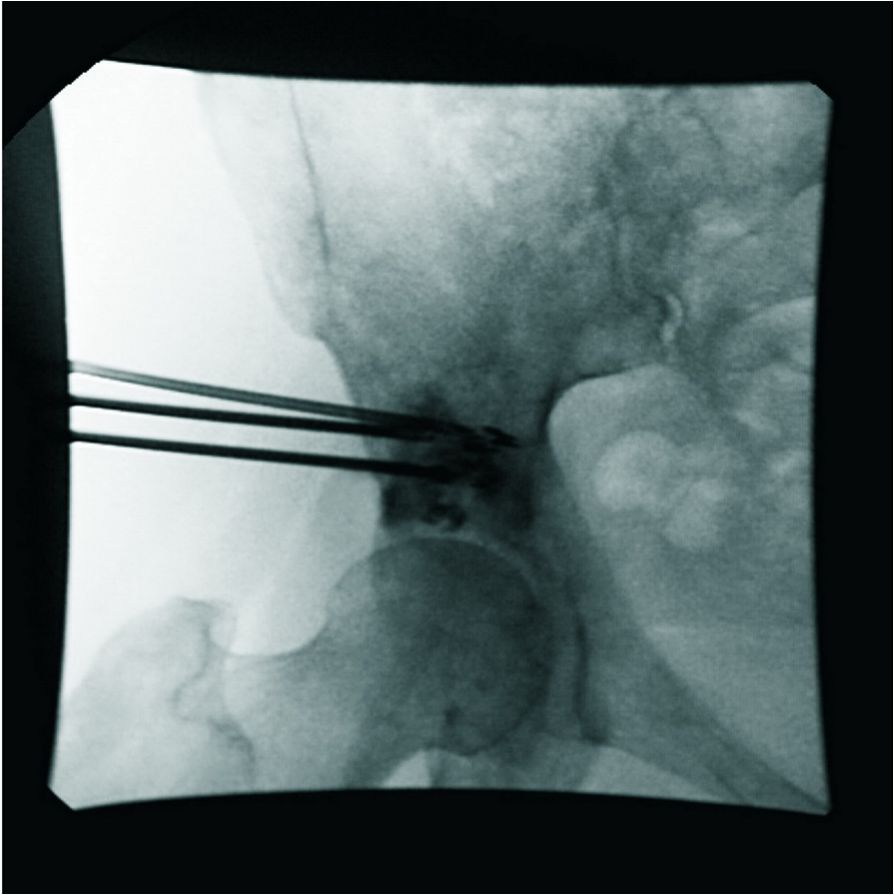
**Percutaneous injection of bone cement combined with **^**125**^**I particle implantation using a DSA machine guide.**

Step 3: The needle core was taken out, and the puncture needle was left in the target area; 5 ml of contrast was injected through the needle, and the contrast medium flow was record by DSA. Polymethyl methacrylate (PMMA) was used as bone cementand was mixed with non-ionic contrast medium during the operation; the injection was conducted under fluoroscopic monitoring to prevent bone cement leakage to the bone (Figure 2).

**Figure 3:**
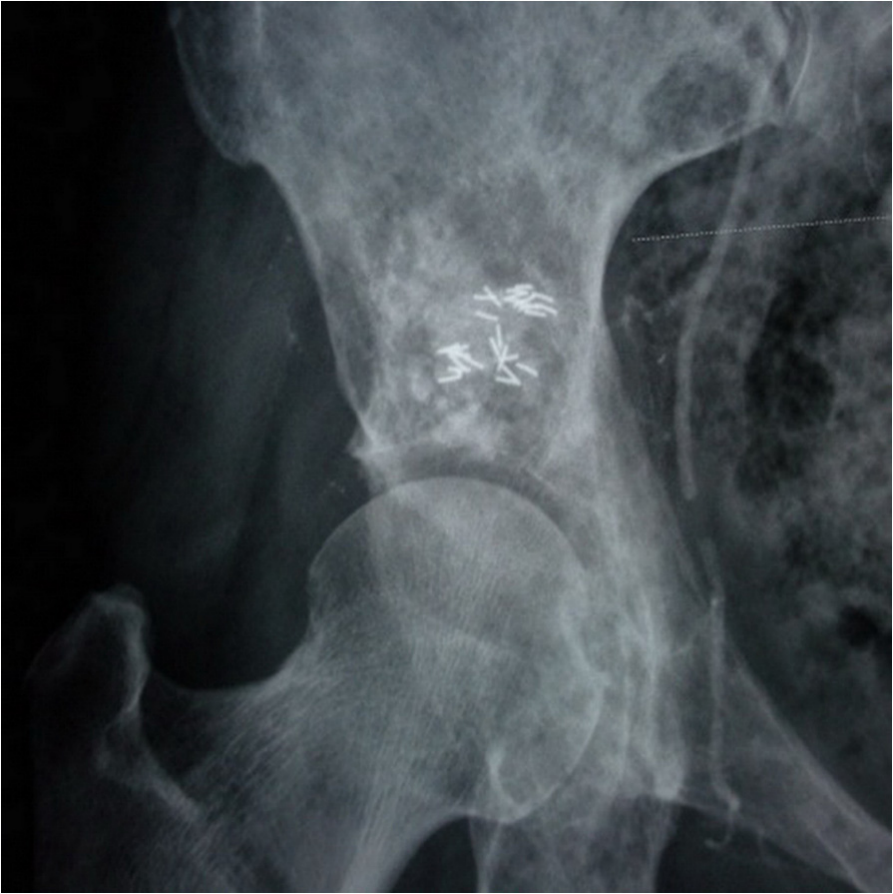
**Postoperative radiographic review showing good **^**125**^**I particle distribution and bone cement filling.**

**Figure 4:**
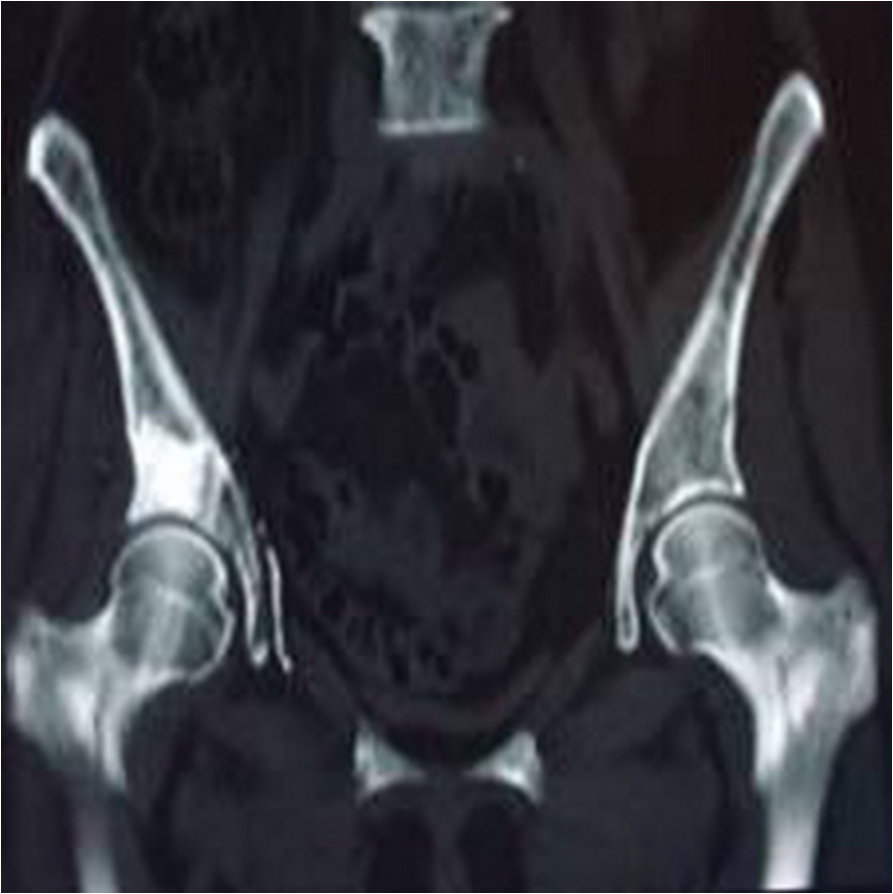
Postoperative CT shows bone cement filling of the cortical bone destruction area.

Step 4: The puncture needle was placed in the cortical bone after injection. The needle core was inserted and rotated to avoid gluing of the bone cement. The needle was pulled out before bone cement hardening. The amount of bone cement injected was 4 to 11 ml; the average amount was 5.5 ml. X-rays (Figure 3) and CT scans (Figure 4) were conducted after 15 to 20 min to determine when the bone cement polymerization reaction was complete. The number of ^125^I particles implanted was 12 to 20; the average number was 16.5.

### Evaluation of therapeutic outcomes

Main outcomes were measured as:

• Postoperative observation of symptoms, change of signs, infection, or bone cement leakage.

• Preoperative and postoperative pain ratings of patients with follow-up and application of narcotic analgesic drugs.

• The Karnofsky Performance Status (KPS) physical status score, the Harris Hip Scale (Harris) score for hip function, and the Visual Analog Scale (VAS) for pain were recorded 1,6 and 12 months before and after the operation in all patients.

### Statistical analysis

Basic statistical analyses were performed using the SPSS 20.0 statistical software (SPSS Inc., Chicago, IL, USA). Categorical data were given as total numbers (and/or relative frequencies). Continuous data were given as mean ± standard deviation. Student’s t-test was performed to compare means between two groups. A value of *P*<0.05 was considered statistically significant.

## Results

All 24 patients’ operations were successful. CT scan results showed bone cement and ^125^I particle distribution in tumor foci in the central region presenting as a mass or diffuse distribution (Figures 3,4). A small amount of PMMA leaked after bone cement injection angioplasty in 15 patients; no clinical symptoms occurred, and there was no need for special treatment.

No periacetabular fractures, increased local lesions, radioactive enteritis, or local wound inflammation occurred during follow-up. Narcotic analgesic drug abuse was alleviated compared to before the operation and disappeared in some patients. Ten patients died in the 3-month follow-up period; one died 3 months after the operation, two died 6 months after the operation, and three died 1 year after the operation. Four other patients died after the 3-year follow-up. They all died of other visceral metastases.

**Figure 5:**
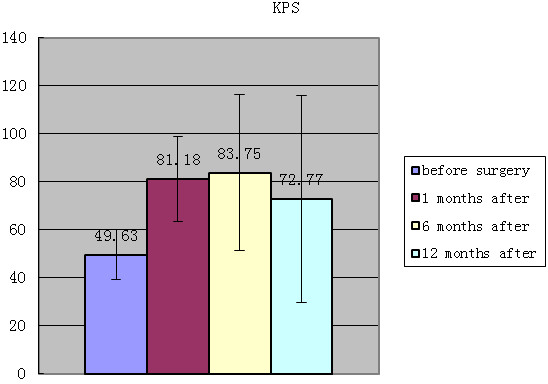
Changes in the Karnofsky Score (KPS).

Continued general improvement was achieved according to the KPS physical status score evaluation. The average KPS scores were 49.63± 10.45, 81.18 ±17.59, and 83.75± 32.60 preoperatively and 1 and 6 months after the operation, respectively. The KPS physical status score decreased 12 months after the operation, with an average score of 72.77± 43.09, showing a worse result in patients with physical conditions; 6 patients died 12 months after the operation (Table 3, Figure 5).

**Table 3:** Changes in the Visual Analog Scale(VAS), Harris Hip Score(Harris), and Karnofsky Score(KPS) scores

**Value**	**Preoperative**	**Postoperative**			***P*value**		
		**1 months**	**6 months**	**12 months**	**P1**	**P2**	**P3**
**KPS**	49.63±10.45	81.18±17.59	83.75±32.60	72.77±43.09	0.000	0.000	0.006
**Harris**	33.94±2.92	71.32±16.57	83.24±32.53	71.70±42.63	0.000	0.000	0.000
**VAS**	7.80±0.42	3.11±0.57	2.03±0.99	3.24±2.35	0.000	0.000	0.000

P1, P2, and P3 are 1,6, and 12 months after the operation, respectively.

**Figure 6:**
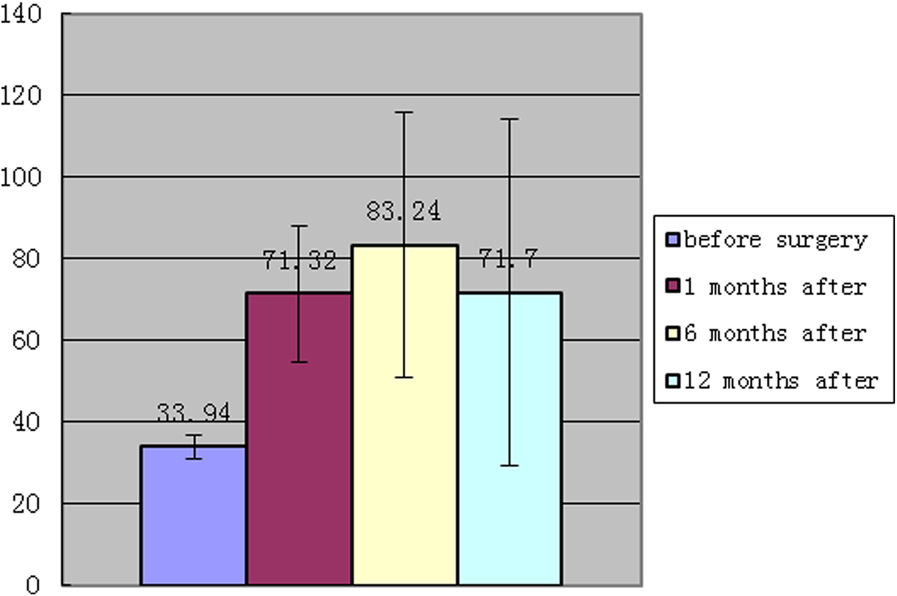
Changes in the Harris Hip Score (Harris).

The Harris hip score reflected the general change in patients in activities of daily living. The score increased from 33.94 ±2.92 before the operation to 71.32± 16.57 1 months after the operation, and further improved to 83.24± 32.53 6 months after the operation. The score decreased to 71.70±42.63 1 year after the operation, but was still higher than the preoperative score (Table 3, Figure 6).

**Figure 7:**
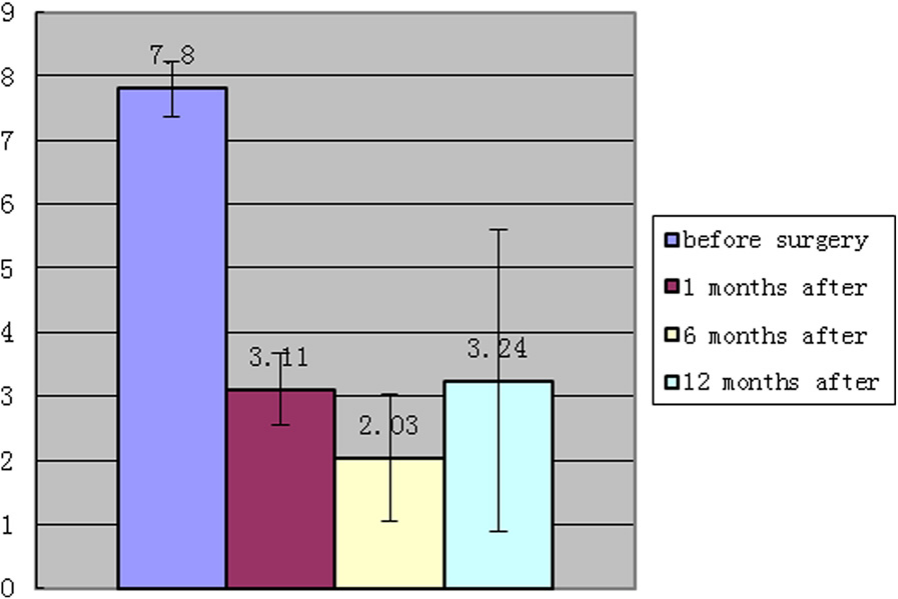
Changes in the Visual Analog Scale (VAS).

Similar trends were seen in patient’s VAS pain score. The pain score decreased from 7.80± 0.42 preoperatively to 3.11± 0.57 1 month postoperatively and continued to decrease to 2.03±0.99 6 months after the operation. This increased to 3.24 ±2.35 1 year after the operation (Table 3, Figure 7).

Statistical analysis results showed statistical significance with *P*< 0.05 for the KPS, Harris, and VAS scores 1, 6, and 12 months before and after the operation (Table 3, Figure 2). In 58% of patients (14 cases) local pain disappeared completely, and pain was alleviated in 42% of patients (10 cases). The pain relief duration was 8.3 months.The pain recurred 3 months after the operation in one patient.

## Discussion

Acetabular metastases are a common and severe complication in advanced malignant disease. The major complications associated with bone involvement are severe pain and bone destruction, all of which restrict mobility and greatly reduce the patient’s quality of life. Periacetabular metastatic tumor treatment involves abalance of survival, function, and quality of life, with limited life expectancy for the patients. Joint function and quality of life are limited, too [20].

Bone destruction in the acetabulum caused by metastatic tumors will cause the femoral head togradually shift toward the medial and top of the acetabulum, the occurrence of pathological fractures, or joint collapse.This can even cause the patient become completely bedridden, increase the chance of pressure sores and hypostatic pneumonia, and seriously influence the patient's quality of life. According to the Enneking partition standard for pelvic tumors [21], periacetabular tumors, which are classified as region lII tumors, are usually widely resected, which has important treatment implications for primary acetabular malignant tumors. However, there is no direct relationship between acetabular resection and the survival rate of patients with metastatic tumors; this is meaningful for alleviating the symptoms and improving function. Effective treatment should relieve the patient’s pain and reconstruct the acetabulum bony structure, enhance the acetabular bearing capacity, and recover walking function [22].

With the injection of bone cement to the acetabular bone destruction zone through percutaneous puncture, we were able to improvethe patients’ functional status and reduce pain. This restored the bone's mechanical properties in the region of the operation immediately, enhanced the local skeletal resistance capacity, reduced the occurrence of pathological fractures, and enhanced the bone’s local stability.

The cytotoxicity of the PMMA bone cement monomer causes tumor cells to dehydrate, solidify, and undergo apoptosis; the polymerization process releases large amounts of heat up to 72 to 78°C. Because of the thermal effects, the tumor tissue and spinal nociceptive nerve endings become necrotic. Solidification of the bone cement increases the bone’s stability and supportive ability.It can reduce pain caused by stimulation of nerve endings, cut off the tumor’s blood supply, and finally cause tumor tissue necrosis.

Cotton [23] first applied the technology to the treatment of acetabular metastases in 1995, reporting 11 cases with percutaneous puncture of the bone using cementoplasty. The pain decreased by 81.8% after 1 to 5days of treatment. Walking function improved in all patients except in one patient,who reported increased pain caused by acetabular fractures. Marcy [13] reported 12 cases of follow-up results of acetabular metastasis in patients 4.6 months after the operation. Pain relief and walking function improved significantly, and one patient had an acetabular fracture 15 days after the operation. Subsequent studies reported satisfactory effects of bone cementoplasty on percutaneous puncture in the treatment of acetabular metastases in the short- and longterm [24,25].

^125^I particle radiotherapy includes external beam therapy (EBRT) and interstitial brachytherapy (IBT). EBRT requires higher radiation doses to achieve the effect becauseperipheral organs affectthe tumor tissue localization. EBRT only works well before tumor metastasis and its efficacy decreases with tumor cell mitosis [16,17]. IBT can maximize the results of irradiation while causing minimal radiation injury of the surrounding normal tissue [18].

^125^I seed implantation is a highly effective treatment for patients with localized cancer. With the advantages of a relatively short half-life, use of low-energy photons with a low risk of complications, and the accuracy and consistency of the seeds, ^125^I seed implantation has been widely used for treatment of all kinds of solid tumors [26,27].

Combined therapy using percutaneous radiofrequency ablation (RFA) and cementoplasty has been shown to be a safe and effective technique for palliative treatment of painful neoplastic bone metastasis [28,29]. In our study of 24 cases of acetabular metastases in patients with percutaneous puncture treated with bone cement combined with ^125^I seed implantation, the pain disappeared completely in 58% (14 cases) of patients and was alleviated in 42% (10 cases) of patients after the operation, with the average duration of pain relief being 8.3 months, except for pain recurrence in one patient 3 months after the operation. Statistical analysis results for the KPS, Harris and VAS scores before and after 1,6, and 12 months showed significant differences. No acetabular fractures or increase of local lesions was found on postoperative follow-up, and the phenomenon of abusing narcotic analgesic drugs was alleviated. However, it is difficult to evaluate narcotic drug use effects systematically, because patients might abuse drugs because of pain caused by tumor metastasis.

Percutaneous puncture of the bone cement combined with ^125^I seed implantation plays an important role in the treatment of metastatic bone tumors. Our early study results of 40 patients with spinal metastases showed good clinical results [30]. The results of applying the combined technology for periacetabular metastatic tumor treatment showed that the combined operation therapy is effective for periacetabular metastatic tumors.

## Conclusions

The combined therapy has antineoplastic characteristics, relieving pain and enhancing skeletal stability. It is a good option to control the development of local tumors and improve patients’ quality of life. Further observation is being conducted to evaluate the prolongation of survival time.

## Competing interests

The authors declare that they have no competing interests.

## Authors’ contributions

JZ and ZY was responsible for whole project design and manuscript writing; JW, JW, PL participated in the surgical operation; HS, KL, and YY were in charge of data analysis and correction of manuscript. All the authors had read and approved the final manuscript.

## Authors’ information

The authors Jinlei Zhang, Zuozhang Yang, Jiaping Wang, Jinde Wang and Pengjie Liu contributed equally to this work, and each is considered as first author.
